# Properties of Saliva

**Published:** 1883-07

**Authors:** 


### PROPERTIES OF SALIVA.

Why has human saliva the power of saccharifying starch-paste, while that of many animals, even herbiferous as the horse, has not? Under the prevalence of atmospheric-germ theories, some have been lately inclined to believe that human saliva owes its power merely to the fact that it is a good medium for the development of amylolytic bacterial organisms. Bechamp, as a result of somewhat extended observations concludes: 1°, that the starch-saccharifying activity of human saliva is not due to chance germs which have entered the mouth from the atmosphere; but 2°, is due to a special ferment more active than diastase; and 3°, produced by the action on the pure secreted saliva of specific microscopic organisms living in the salivary glands and in the mouth-cavity of man. The pure parotid saliva of horse or dog does not convert starch-paste into copper-oxide-reducing substances, nor does it acquire this power when exposed to the air, or when gently warmed along with scrapings from the tongues of those animals; but when scrapings from the inside of the human mouth are added to it, it soon becomes a very efficacious agent for the saccharification of starch.—*Arch. physiol. norm. Path.*

